# The role of DJ-1 in anti-apoptosis

**DOI:** 10.1186/1750-1326-7-S1-L16

**Published:** 2012-02-07

**Authors:** Haigang Ren, Kai Fu, Jun Fan, Guanghui Wang

**Affiliations:** 1 Laboratory of Molecular Neuropathology, Department of Pharmacology, Soochow University College of Pharmaceutical Sciences, Suzhou, Jiangsu 201203, China; 2 Laboratory of Molecular Neuropathology, Key Laboratory of Brain Function and Diseases and School of Life Sciences, University of Science & Technology of China, Chinese Academy of Sciences. Hefei, Anhui 230027, China

## Background

DJ-1 is a protein in association with Parkinson’s disease (PD) and cancers. DJ-1 has been reported to exhibit both cytoplasmic and nuclear distribution (Bonifati *et al.*, Science, 2003; Nagakubo *et al.*, BBRC, 1997). It functions in multiple pathways to affect cell survival. It suppresses the JNK signaling pathway in cytoplasma (Mo *et al.*, Cell Death Differ, 2008) and interacts with Daxx and sequesters it within the nucleus, preventing the initiation of apoptotic signaling (Junn *et al.*, PNAS, 2005). In contrast to its functions in cell survival, deletions or loss of function point mutations in DJ-1 are reported to be responsible for recessive early-onset Parkinson’s disease (PD) (Bonifati *et al.*, Science, 2003). The most commonly studied PD-associated mutant, L166P, is reported to be unstable and to mislocalize to the mitochondria, leading to a loss of the cytoplasmic function of DJ-1 (Bonifati *et al.*, Science, 2003). Although lines of evidence suggest that a high expression of DJ-1 enhances cell survival and loss of DJ-1 function is associated with PD, the detailed mechanisms are still not fully understood.

## Results

In our studies, we show that DJ-1 functions in multiple ways to affect cell survival. It inhibits TRAIL-induced apoptosis by blocking Fas-associated protein death domain (FADD)-mediated pro-caspase-8 activation. Wild-type DJ-1, but not the PD-associated mutant L166P, binds to FADD to inhibit the formation of the death-inducing signaling complex (DISC). DJ-1 competes with pro-caspase-8 to bind to FADD at the death effector domain (DED), thereby repressing the recruitment and activation of pro-caspase-8 to the active form of caspase-8, suggesting that DJ-1 protects against TRAIL-induced apoptosis through the regulation of DISC formation. DJ-1 and DJ-1(L166P) have potential roles in mitochondria. DJ-1(L166P) but not DJ-1 co-localizes with and interacts weakly with Bcl-X_L_, whereas both DJ-1 and DJ-1(L166P) increase in mitochondria in response to ultraviolet B (UVB) irradiation and have increased binding to Bcl-X_L_. Moreover, DJ-1 but not DJ-1(L166P) stabilizes Bcl-X_L_ by inhibiting its ubiquitination in response to UVB irradiation. Besides its role in cytoplasma, DJ-1 exerts its cytoprotection through inhibiting nuclear p53. DJ-1 interacts with p53 *in vitro* and *in vivo*. Overexpression of DJ-1 decreases the expression of Bax and inhibits caspase activation, while knockdown of DJ-1 increases Bax protein level, and accelerates caspase-3 activation and cell death induced by UV exposure. A sumoylation-deficient mutant of DJ-1, DJ-1(K130R), shifts from nucleus to cytoplasm and fails to repress p53 transcriptional activity on Bax promoter.

## Conclusion

Our data provide evidence that DJ-1 plays important roles in anti-apoptosis by its function in multiple pathways.

